# Recent Advances in Dromedary Camels and Their Products

**DOI:** 10.3390/ani12020162

**Published:** 2022-01-11

**Authors:** Mohammed Gagaoua, Amira Leila Dib, El-Hacene Bererhi

**Affiliations:** 1 Food Quality and Sensory Science Department, Teagasc Food Research Centre, Ashtown, D15 KN3K Dublin, Ireland; 2 Gestion Santé et Productions Animales Research Laboratory, Institut des Sciences Vétérinaires El-Khroub, Université Frères Mentouri Constantine 1, Constantine 25000, Algeria; dibamira@hotmail.com (A.L.D.); brerhihacene@yahoo.fr (E.-H.B.)

Dromedary camels or, more specifically, one-humped camels (*Camelus dromedarius*), are described as having a high productive potential, and for centuries, they have been used by people (namely nomads) in arid and hot regions as multipurpose animals for physical labor, transport, the production of milk, meat, wool, hair, and skin, and for racing and tourism. Camels are widespread in the arid regions of the east of Asia to the North of Africa, with an overall population that is estimated to be around 30 million animals. Due global climate changes that are characterized by a continuous increase in desertification, changing and high temperatures as well as drought, it is important to reconsider the dromedary camel as one of the most adapted and sustainable animals that can be used to overcome such challenging environmental conditions. Indeed, several recent reports have highlighted that due to the advancement of the desert world, the dromedary camel will be the main animal that survives as the best source livestock for future agriculture and for the animal production sector, mainly by playing a significant role in achieving the objectives of the Sustainable Development Goals. However, compared to other livestock species, dromedaries have received less attention by the scientific community. In this context, a better understanding of the behavioral and physiological properties of dromedary camels is a prerequisite to facilitate ideal way to harness their natural advantages, especially under intensive farming systems. Thus, this Special Issue aimed to gather studies on recent advances in dromedary camels and their products (milk and meat).

This Special Issue addresses interdisciplinary research and includes four manuscripts (one review paper and three research articles), all of which are important contributions to this topic and that were prepared by distinguished camelids experts in this area. The first manuscript that is a comprehensive review summarizes the current knowledge on camel milk and its processing [1]. The global camel milk market is undergoing significant changes that are mainly based on two structural innovations. First, there is an emergence of intensive production systems under an entrepreneurial approach that seems to be significantly disconnected from the pastoral dynamics. Second, the periurban camel production systems have been developed, which significantly contribute to the urban camel milk supply while maintaining strong links with the pastoral economy. In this manuscript, the authors reviewed the available knowledge from the literature concerning the processing of camel milk into different products and focused on (i) pasteurized milk, (ii) fermented milk, (iii) camel cheese, and (iv) camel milk powder. Further, the authors briefly discussed the available knowledge on other products, such as yogurt, butter, and the sweet and non-alimentary processing of camel milk [1]. Compared to milk processing from other dairy species, the modernized processing of camel milk appears to be recent and requires more innovations in the future. Further, the authors pointed out that the technologies that are currently being applied for the transformation of cow milk into fermented or pasteurized milk or into other products such as cheese, yogurt, or powder are not fully appropriate for camel milk. Hence, adaptations based on fundamental and applied approaches are required.

The second study investigated a very important societal and public issue, animal welfare, which is becoming an increasingly significant component of contemporary global livestock production due to its recognized effects on public health and the sustainability of food production [2,3,4]. Therefore, Menchetti et al. [5] aimed in their original study to achieve several objectives, these being (i) applying the first protocol assessing camel welfare previously developed by the same group [6], (ii) developing a system to score the defined welfare measures, (iii) producing overall assessment indices, and (iv) classifying the animal units with a focus on pens based on their welfare level. Based on an important number of camel welfare measures (*n* = 105) collected from 76 pens from a camel market, the indices for thirst, disease and physical injuries, feeding and watering management, the presence of a shelter, body condition score, and bedding cleanliness were the main measure drivers considered for pen classification. More specifically, the authors proved through this study that although further validation is needed, a good feeding index (including prolonged thirst and hunger), was the most critical measure, while a good health index, including measures related to the absence of injuries, disease, and pain, seemed to be less problematic. Finally, the preliminary model of this trial showed the possibility to accurately identify the major welfare concerns of dromedary camels kept at the market, hence allowing possible corrective actions to be suggested in the future.

The third study was also designed in the context of animal welfare and, more specifically, to study the camel performance and the breeding behaviour of male dromedaries during the rutting season [7]. Under intensive farming systems, dromedaries are kept in captive conditions and, similarly, during the rutting season, the males are kept tied with ropes in small pens and/or kept in single stalls with the objective of avoiding aggression against other males or humans. However, this housing system can have a negative effect on the welfare of the dromedary camels and might also have a negative effect on sexual activity, leading to significant impacts on animal performance and reproduction. This study then aimed to study the effect of different housing management systems on the maintenance, posture and sexual behaviors, blood metabolites, and hormonal balance of captive male dromedaries. It was designed to overcome the drawbacks of captive conditions during the rutting season with the objective of accomplishing an ultimate housing system for male dromedaries (i) to maximize animal reproductive performance, (ii) to reduce sexually associated aggressiveness, and consequently, (iii) to improve camel welfare. The male dromedaries who were housed in groups and who were allowed to walk around had greater percentages of ruminating, standing, walking, and sexual-related behaviors compared to the dromedaries who were housed individually or who were tied. Moreover, the metabolites measured from blood serum and certain hormone concentrations were significantly affected by movement control. This study concluded that social interaction is important to camels for maintaining the psychological, physical, and sexual behaviors of camel dromedaries [7]. Thus, allowing walking-around exercise for captive camels can significantly improve their metabolic status while decreasing the stress effects of captive housing. From these findings, one can conclude that housing systems that guarantee physical activity and social interaction are more suitable for male dromedaries during the rutting season.

The last study in the context of zoonosis and camel health investigated the use of a molecular characterization of the tick-borne hemoparasites in dromedary camels in the Al Dhafra region of Abu Dhabi, United Arab Emirates (UAE) [8]. This is the first molecular investigation study on tick-borne pathogens in dromedary camels from the UAE. It highlights, although with a very low number of samples, the existence of a possible risk of infection or contamination for humans working closely with the infected camels. Many species of arthropods and parasites are known to affect dromedary camels, including ticks, and there is a risk of transmitting pathogens between humans and animals. Thus, the authors searched for the presence of blood parasites in 93 camels who had been diagnosed with acute clinical signs, from which 72 ticks were collected and characterized in-depth. It was determined that the 72 ticks that were collected in this study were identified as *Hyalomma dromedarii* species. They were also all found to be negative for blood pathogens. The DNA analyses on the dromedary blood samples was positive for tick-transmitted pathogens in 15 heads, including 15 *Anaplasma phagocytophilum*, *Coxiella burnetii*, and *Babesia*/*Theileria* spp. Only one coinfection of *A. phagocytophiulm* and *C. burnetii* was detected in this study.

This Special Issue grouped some of the most recent studies on camelids related to animal welfare, zoonosis, reproduction, and products with a focus on camel milk and its processing and attempts to contribute to the growth of the scientific knowledge in this camelid research area. We hope that this Special Issue will attract the interest of not only animal production researchers and camelid experts but also the attention of students and breeders who have an interest in dromedary camels by inspiring them to conduct research in the areas that were not covered in this Special Issue. We would like to thank all of the authors who generated the data and who contributed to this Special Issue by sharing their scientific knowledge and original studies and to all of our colleagues who reviewed, read, and dispersed the information contained within these studies. We sincerely hope that readers will find this Special Issue motivating and informative.

## Data Availability

Not applicable.
